# Scale for Body Image Concerns During the Perinatal Period – Adaptation and validation

**DOI:** 10.1192/j.eurpsy.2024.355

**Published:** 2024-08-27

**Authors:** A. T. Pereira, B. Barbosa, R. Lima, A. I. Araújo, C. Marques, D. Pereira, A. Macedo, C. Pinto Gouveia

**Affiliations:** ^1^ Institute of Psychological Medicine; ^2^Faculty of Medicine, University of Coimbra, Coimbra, Portugal

## Abstract

**Introduction:**

The perinatal period may intensify weight and body image concerns. Due to its specifics, the traditional body image scales are inaccurate in the perinatal period (Fuller-Tyszkiewicz et al. 2013). The Body Image Concerns During Pregnancy (Uçar et al. 2018) was developed to measures this cognitive-emotional variable in pregnancy.

**Objectives:**

To analyze the psychometric properties of the Portuguese adapted (both for pregnancy and postpartum) version of the Body Image Concerns during the Perinatal Period (BICPP), namely its construct validity and the internal consistency.

**Methods:**

A sample of 346 women recruited through social media and Family Health Units, assessed in the second trimester of pregnancy (mean gestational age=28.11±7.67 weeks) and after delivery (baby’s age 4.37±2.87 months), completed a survey including the Portuguese BICPP.

The total sample was randomly divided into two sub-samples: sample A (n=173) was used to perform an exploratory factor analysis/EFA; sample B (n=173) to perform a confirmatory factor analysis/CFA.

**Results:**

EFA resulted in four components. CFA revealed that the second-order model with four factors presented good fit indexes (X2/df=2.4141; CFI=.9195; GFI=.948; TLI=.9028; GFI=.8181; RMSEA=.0807). BICPP Cronbach alphas was α=.936; for F1 Concern about future weight and image, F2 Concern with the new body image, F3 Social avoidance and concern and F4 Concern with appearance were .922, .930, .809, .807, respectively.

**Conclusions:**

This psychometric study provides evidence for the validity and reliability of the Portuguese version of BIC-Perinatal Period, which will be used in an ongoing research project on the relationship between eating, depressive and anxiety disorders in the perinatal period.

**Disclosure of Interest:**

None Declared

